# The chromosome-level holly (*Ilex latifolia*) genome reveals key enzymes in triterpenoid saponin biosynthesis and fruit color change

**DOI:** 10.3389/fpls.2022.982323

**Published:** 2022-08-22

**Authors:** Ke-Wang Xu, Xue-Fen Wei, Chen-Xue Lin, Min Zhang, Qiang Zhang, Peng Zhou, Yan-Ming Fang, Jia-Yu Xue, Yi-Fan Duan

**Affiliations:** ^1^Key Laboratory of National Forestry and Grassland Administration on Subtropical Forest Biodiversity Conservation, Co-innovation Center for Sustainable Forestry in Southern China, College of Biology and the Environment, Nanjing Forestry University, Nanjing, China; ^2^College of Horticulture, Academy for Advanced Interdisciplinary Studies, Nanjing Agricultural University, Nanjing, China; ^3^Jiangsu Academy of Forestry, Nanjing, China

**Keywords:** Aquifoliales, whole-genome sequencing, genome evolution, pentacyclic triterpenoid saponins, anthocyanidins biosynthesis genes, holly

## Abstract

The *Ilex* L. (hollies) genus of Aquifoliaceae shows high species diversity in tropical and subtropical regions of Asia and South America. Throughout the range of the genus, *Ilex* species have been widely used in beverage and medicine production and as ornamentals. Here, we assembled a high-quality, chromosome-level genome of *Ilex latifolia*, which has extremely high economic value because of its useful secondary metabolite production and the high ornamental value of its decorative red berries. The 99.8% genome sequence was anchored to 20 pseudochromosomes, with a total length of 766.02 Mb and a scaffold N50 of 33.45 Mb. Based on the comparative genomic analysis of 14 angiosperm species, we recovered *I. latifolia* as the sister group to all other campanulids. Two whole-genome duplication (WGD) events were identified in hollies: one shared ancient WGD in the ancestor of all eudicots and a recent and independent WGD in hollies. We performed a genome-wide search to screen candidate genes involved in the biosynthesis of pentacyclic triterpenoid saponins in *I. latifolia*. Three subfamilies of CYP450 (CYP71A, CYP72A, and CYP716A) appear to have expanded. The transcriptomic analysis of *I. latifolia* leaves at five developmental stages revealed that two CYP716A genes and one CYP72A gene probably play important roles in this biosynthetic pathway. In addition, we totally identified 12 genes in the biosynthesis pathways of pelargonidin and cyanidin and observed their differential expression in green and red fruit pericarps, suggesting an association between pelargonidin and cyanidin biosynthesis and fruit pericarp color change. The accumulation of pelargonidin and cyanidin is expected to play an important role in the ornamental value of *I. latifolia*. Altogether, this study elucidated the molecular basis of the medicinal and ornamental value of *I. latifolia*, providing a data basis and promising clues for further applications.

## Introduction

*Ilex* L. (the hollies) is a genus of trees, shrubs, and (rarely) climbers within the Aquifoliaceae family that contains more than 600 species with an irregular cosmopolitan distribution, in which most species occur in tropical and subtropical regions of South America and Asia (Loizeau et al., 2016). Hollies have high economic and ornamental value. The leaves of over 60 species of this genus are used in beverages (Loizeau et al., 2016) such as Paraguay tea (mate tea) made from *Ilex paraguariensis* A. St.-Hil., which is drunk throughout South America (Bracesco et al., 2011), and the black drink, or “Cassena,” made from *Ilex vomitoria* Aiton, which has been used by certain native North Americans (Folch, 2021). Many *Ilex* species (e.g., American holly, *Ilex opaca* Aiton; Japanese holly, *Ilex crenata* Thunb.; and *Ilex purpure* Hassk. in China) are widely grown in parks and gardens throughout the world for their foliage and decorative berries (e.g., on Christmas trees) and hold an important position in gardens worldwide. However, genetic research on holly species has mainly focused on evaluating their genetic diversity by identifying molecular markers, determining phylogenetic relationships, and revealing speciation and lineage diversification until now (Gottlieb et al., 2005; Selbach-Schnadelbach et al., 2009; Manen et al., 2010; Shi et al., 2016; Xu et al., 2021; Yao et al., 2021). The weak foundation of genome-wide research not only for the entire family Aquifoliaceae but also for the order Aquifoliales is not equal to the importance of these groups.

**FIGURE 1:**
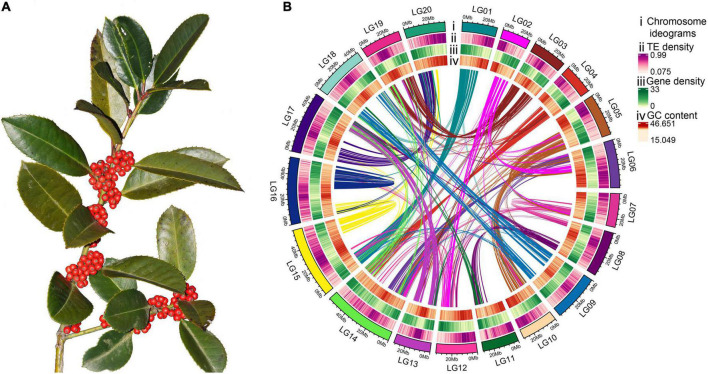
Morphology and high-quality genome assembly of *Ilex latifolia*. **(A)** Fruiting branches of *I. latifolia* showing its ornamental berries. **(B)** The genome features across 20 chromosomes of *I. latifolia*. From outermost to innermost, the circles show chromosome ideograms; TE, transposable element (long and short interspersed nuclear elements) density; gene density; GC content and colinear blocks in the genome.

*Ilex latifolia* Thunb. is a subtropical evergreen tree native to China and Japan (Figure 1A). In addition to its ornamental functions, the tender leaves of this plant can be processed into a specific kind of tea known as Kudingcha (Sun et al., 2011). Kudingcha has a slight bitter taste, and its Chinese name clearly reflects its bitter flavor; it is known as a very healthy drink and is a traditional Chinese medicine with a long history in southern China because the young leaves of *I. latifolia* contain pentacyclic triterpenoid saponins and flavonoids, which have blood lipid- and blood pressure-lowering, detoxification and cancer-combating effects (Li et al., 2013; Yang et al., 2015). Because of these benefits to human health, Kudingcha has become the plant-based beverage with the highest production in China, second only to tea [from young leaves of *Camellia sinensis* (L.) Kuntze]. However, the genome of *I. latifolia* has not been sequenced, and candidate genes associated with some important active ingredients of Kudingcha and the ornamental traits of the genus have yet to be identified. The lack of transcriptional and genomic information on *I. latifolia* has greatly limited genetic and breeding research on this species and its closely related congeners in the genus. Therefore, more detailed molecular and genomic resources are still needed to investigate the genomic signatures of holly species.

Recently, Yao et al. (2022) assembled the first holly genome of a deciduous species [*Ilex polyneura* (Hand.-Mazz.) S.Y. Hu] and performed a population genomics study to clarify biogeographic ambiguities but did not focus on the molecular mechanisms underlying the medicinal and ornamental value of hollies. In this study, we therefore sequenced and assembled a chromosome-level genome for *I. latifolia* using Nanopore technology combined with Hi-C scaffolding and analyzed its evolutionary and genomic features. In addition, we performed a transcriptomic analysis of its leaves at multiple developmental stages to identify critical genes involved in the biosynthesis of pentacyclic triterpenoid saponins. Candidate genes associated with fruit anthocyanin biosynthesis were also identified *via* a combined analyses of transcriptomic data at two different developmental stages. This study provides the second comprehensive *Ilex* genome reported to date and provides insight into the biosynthesis of pentacyclic triterpenoid saponins and the coloration of fruits in economic and ornamental tree plants.

## Materials and methods

### Sample collection and Illumina and Nanopore sequencing

In this study, all materials used for genome sequencing were collected from an adult *I. latifolia* plant growing on the campus of Nanjing Forestry University. Approximately 500 mg of tissue was dissected and stored in liquid nitrogen until it was delivered in dry ice. Total genomic DNA extraction was performed by using a sodium dodecyl sulfate (SDS)-based method before purification with chloroform for Illumina and Nanopore sequencing. For Illumina sequencing, DNA was sonicated to a fragment size of 500 bp with an ultrasonicator, and the library was then prepared by using an NEB Ultra DNA library prep kit (NEB, United Kingdom) according to the manufacturer’s instructions. The paired-end sequencing of the libraries was performed on a NovaSeq 6000 system. A total of 88.62 Gb of raw data were obtained, and unpaired reads, low-quality reads, connector contamination, and duplicated reads were filtered to obtain clean data (Supplementary Table 1).

For Nanopore sequencing, 2 μg of gDNA was repaired using the NEB Next FFPE DNA Repair Mix kit (M6630, United States) and subsequently processed using the ONT Template prep kit (SQK-LSK109, United Kingdom) according to the manufacturer’s instructions. The large-segment library was premixed with loading beads and then pipetted into a previously used and washed R9 flow cell. The library was sequenced on the ONT PromethION platform with the corresponding R9 cell and ONT sequencing reagent kit (EXP-FLP001.PRO.6, United Kingdom) according to the manufacturer’s instructions.

### Estimation of genome size, heterozygosity, and repeat content

Before genome assembly, the read information obtained by sequencing was subjected to K-mer analysis. The occurrence of k-mers was counted with Jellyfish (Marçais and Kingsford, 2011). The general features of the genome, including its repeat contents, heterozygosity rates, and genome size, were estimated with GenomeScope (Vurture et al., 2017).

### *De novo* genome assembly of Nanopore reads and assembly assessment

Nanopore reads were initially corrected using Canu (Koren et al., 2017) and then used as input data for SMARTdenovo^1^ assembly. After completing the initial assembly, Racon (Vaser et al., 2017) and Pilon (Walker et al., 2014) were used to calibrate and polish the assembled reference genome by using Nanopore and Illumina data. In addition, CEGMA v2.5 (Parra et al., 2007) and BUSCO v2.0 (Simão et al., 2015) were used to assess the genome completeness and gene set completeness of the draft genome sequences (Supplementary Tables 2, 3).

### Hi-C chromosome assembly

The adapter sequences of the raw Hi-C reads were trimmed, and low-quality paired-end reads were removed to obtain clean data. Then, the clean reads were aligned to the assembled results using BWA v0.7.10-r789 (Li and Durbin, 2009). Invalid read pairs, including dangling-end, self-circularized, religated and dumped products, were filtered, and valid interaction read pairs were identified and retained from the uniquely mapped paired-end reads with HiC-Pro v2.8.1^2^ (Servant et al., 2015). These corrected scaffolds were then clustered, ordered, and oriented onto chromosomes using LACHESIS (Belton et al., 2012) with the following parameters: CLUSTER_MIN_RE_SITES = 5, CLUSTER_MAX_LINK_DENSITY = 2, CLUSTER NONINFORMATIVE RATIO = 2, ORDER MIN N RES IN TRUNK = 5, ORDER MIN N RES IN SHREDS = 100. Finally, placement and orientation errors exhibiting obvious discrete chromatin interaction patterns were manually adjusted. The total length of pseudochromosomes consisted of 93.25% of all genome sequences (Supplementary Figure 1 and Supplementary Tables 4, 5).

### Genome annotation

The *I. latifolia* genome was annotated using genomic sequences as well as repeated sequences, gene structure information, non-coding RNAs, pseudogenes, and gene function information. For repeated sequences, a *de novo* repeat library was initially constructed using LTR FINDER v1.05 (Xu and Wang, 2007) and Repeat Scount v1.0.5 (Price et al., 2005). The repeat library was classified by PASTEClassifier (Hoede et al., 2014) and then merged with Repbase (Jurka et al., 2005). Repeated sequence annotation was conducted according to the homolog method by RepeatMasker (Chen, 2004) using the merged database (Supplementary Table 6). *De novo* repeat annotation was performed using RepeatModeller (Flynn et al., 2020). Three methods were selected to annotate gene structures. First, Genscan (Burge and Karlin, 1997), AUGUSTUS (Stanke et al., 2006), GlimmerHMM (Majoros et al., 2004), Gene ID (Alioto et al., 2018), and SNAP (Korf, 2004) were applied for *de novo* prediction according to the *I. latifolia* genome. Second, the protein sequences of three related species were selected for homologous annotation using GeMoMa (Keilwagen et al., 2019). Third, transcript annotations were performed based on the RNA sequencing results using HISAT (Kim et al., 2015), StringTie (Pertea et al., 2015), TransDecoder, GeneMarkS-T (Tang et al., 2015), and PASA (Campbell et al., 2006). Finally, all acquired data based on the three predictions were combined and revised using EVidenceModeler (EVM) (Haas et al., 2008) and PASA (Campbell et al., 2006; Supplementary Figure 2 and Supplementary Tables 7, 8). The annotations of the non-coding RNAs included microRNAs (miRNAs), ribosomal RNAs (rRNAs), and transfer RNAs (tRNAs). Infenal v1.1 (Nawrocki and Eddy, 2013) was used to annotate miRNAs and rRNAs based on the miRbase (Kozomara et al., 2019) and Rfam (Griffiths-Jones et al., 2005) databases, respectively. tRNAscan-SE (Lowe and Eddy, 1997) with the “-E -H” option was applied to detect the tRNA sequences. Pseudogene homolog sequences were subjected to BLAST searches using GenBlastA (She et al., 2009). The non-mature termination codes and frameshift mutations of pseudogenes were analyzed and annotated using GeneWise (Birney et al., 2004). Gene functions were annotated *via* protein databases, including the NCBInr, euKaryotic Orthologous Groups (KOGs) (Koonin et al., 2004), Gene Ontology (GO) (Ashburner et al., 2000), Kyoto Encyclopedia of Genes and Genomes (KEGG) (Kanehisa and Goto, 2000), and SWISS-PROT/TrEMBL (Boeckmann et al., 2003) databases, using protein sequences whose structures had been annotated (Supplementary Table 9).

### Comparative genomics and genome evolution analyses

Orthologous gene clusters of *I. latifolia* and 13 other angiosperms were identified using OrthoFinder (Emms and Kelly, 2019) with the default parameters. A total of 13,615 homologous groups were identified in *I. latifolia*, and 1,002 low-copy orthologous genes were identified in this set. The protein sequences of low-copy orthologous genes were aligned using MUSCLE v3.8.31 (Edgar, 2004) and were then used to build a highly supported maximum likelihood (ML) tree of 14 angiosperm species with the JTT + F + R5 best-fit model using IQ-TREE v1.6.11 (Nguyen et al., 2015). To further estimate the divergence times of *I. latifolia* and the other 13 angiosperm species, the MCMCTree program included in PAML v4.9i (Yang, 2007) was applied to calculate their divergence times. Four calibration points were selected from TimeTree^3^ as normal priors to reduce age, referencing speciation times of 168–194 Mya for the split of *I. latifolia* and *Amborella trichopoda*, 148–173 Mya for that of *I. latifolia* and *Piper nigrum*, 110–124 Mya for that of *I. latifolia* and *Arabidopsis thaliana*, and 88–106 Mya for that of *I. latifolia* and *Panax notoginseng*.

The analysis of the expansion and contraction of orthologous gene families in *I. latifolia* and 13 other angiosperm species was performed using the software CAFE 5 (Mendes et al., 2021). WGD software (Zwaenepoel and Van de Peer, 2019) was used for the analysis of synonymous substitutions per synonymous site (Ks) value-based paralog age distributions. All potential paralogs were detected *via* all-vs.-all protein sequence BLAST searches using BLASTP with an *e*-value cut-off of 10^–10^, and the MCL package was then used for gene family construction. MAFFT (Rozewicki et al., 2019) was used to align each family. Then, gene families (with n members) of n*(n-1)/2 > “max pairwise” were removed, and a phylogenetic tree was built for each gene family using FastTree (Price et al., 2009). Ks values were calculated using the maximum-likelihood method in the CODEML program of the PAML v.4.4c package (Yang, 2007). Finally, we performed mixture modeling for all possible WGD inferences using the BGMM method. The JCVI (Tang et al., 2008) and Minimap packages (Li, 2018) were used for syntenic visualization. The WGDI package (Sun et al., 2021) was used for collinear anchor pair identification and analysis. All syntenic blocks were identified using the improved collinearity pipeline in WGDI with the “*p*-value = 0.05” setting, and the Ks value for each anchor pair gene located in a syntenic block was calculated using the Ks pipeline in WGDI. The Ks dotplot of all anchor pairs was obtained by applying the block pipeline in WGDI. The KsPeaks pipeline in WGDI was used for the distribution analysis of the Ks median value for each syntenic block. Finally, all of the above results from the Ks distributions were summarized in one picture by plotting with the ggplot2 package.

### Transcriptome sequencing and evolutionary analysis of gene families in *Ilex*

The leaves of *I. latifolia* and *I. cornuta* at five developmental stages and the fruits of *I. latifolia* at two developmental stages (green and red) were collected from plants cultivated on the campus of Nanjing Forestry University. Three replicate samples were selected for each stage. All samples were frozen in liquid nitrogen immediately after harvesting. The RNA of the samples was extracted using the RNA Plant Plus Kit (Tiangen, DP473) according to the manufacturer’s protocol. Illumina RNA-Seq libraries were prepared and sequenced on a HiSeq 2500 system following the manufacturer’s instructions (Illumina, United States). Raw reads were trimmed to remove adaptors, and short reads (<100 bp after trimming) were discarded. The TopHat2 package (Kim et al., 2013) was used to map clean reads to the genome with the default parameters. Transcripts were assembled using Cufflinks (Trapnell et al., 2012), and TransDecoder software^4^ was used for annotation. Gene expression levels were calculated and normalized using the FPKM method, followed by the application of RSEM software (Li and Dewey, 2011). Gene expression heatmaps were generated using TBtools (Chen et al., 2020).

HMMER (Finn et al., 2011) and BLASTP (Camacho et al., 2009) were used to identify putative P450 gene families from the protein sequences of *I. latifolia* and *I. cornuta*. The Hidden Markov Model (HMM) database with the seed file (PF00067) of the P450 genes and the P450 family proteins of *Arabidopsis thaliana* were obtained from the *Arabidopsis* Information Resource (TAIR).^5^ The filtered sequences were further subjected to BLAST searches using the NCBI database^6^ with a cut-off *E*-value of 10^–5^. Sequences annotated as P450 members in *I. latifolia* and *I. cornuta* were collected and aligned with those from *Amborella trichopoda*, *Arabidopsis thaliana*, *Oryza sativa*, and *Panax notoginseng* using the ClustalW program (Oliver et al., 2005). An unrooted maximum-likelihood phylogenetic tree was then constructed using IQ-TREE (Nguyen et al., 2015) and visualized using Figtree^7^ and iTOL (Letunic and Bork, 2021). The identification of genes in the anthocyanin synthesis pathway following the same methods applied to the P450 gene family.

## Results

### Genome sequencing, assembly, and annotation

In this study, 71,218,217,963 k-mers were produced, and the peak depth of the k-mers was 89 (Supplementary Figure 3). The whole-genome size was approximately 772.55 Mb, which was close to the genome size estimated from flow cytometry (Su et al., 2020). The calculated repeat and heterozygosity rates were 47.59 and 0.85%, respectively (Supplementary Table 10). To obtain a high-quality genome, a combination of 88.6 G Illumina paired-end reads (120×), 82.4 G Nanopore single-molecule long reads (N50 = 33.1 Kb, 108×), and 83.8 G of Hi-C sequencing data (110×) were used for assembly. After genome assembly, polishing, and redundancy elimination, an initial assembly of 765.94 Mb with 844 contigs was obtained for the *I. latifolia* genome (contig N50 = 1.46 Mb), which was further assembled into 344 scaffolds (scaffold N50 = 33.4 Mb) (Supplementary Table 11). Altogether, 764.44 Mb (99.8%) of the assembly could be anchored to 20 pseudochromosomes (Figure 1B), and the genome-wide interaction heatmap showed high-quality grouping and ordering results based on the Hi-C data (Supplementary Figure 1).

Genome annotation indicated that the *I. latifolia* genome contained 406 Mb (53.0%) of repetitive sequences, among which LTR elements were the most abundant components (29.4%) (Supplementary Table 6). Regarding protein-coding genes, *ab initio* predictions incorporating the homology-based method and transcriptome data identified 35,218 genes in the *I. latifolia* genome, with an average coding-sequence length of 1.55 Kb and an average of 5.2 exons per gene. Among all annotated genes, 24,498 (69.6%) were supported by transcriptomic data, and 30,586 (86.8%) genes could be functionally annotated (Supplementary Figures 4, 5). Our annotation captured 1,328 (92.22%) complete benchmarking universal single-copy ortholog (BUSCO) genes, suggesting that our assembly achieved a high level of genome completeness. In addition to protein-coding genes, we identified 448 transfer RNAs, 314 ribosomal RNAs, and 127 microRNAs.

### Phylogenetic position of Aquifoliales and whole-genome duplications in *Ilex latifolia*

**FIGURE 2:**
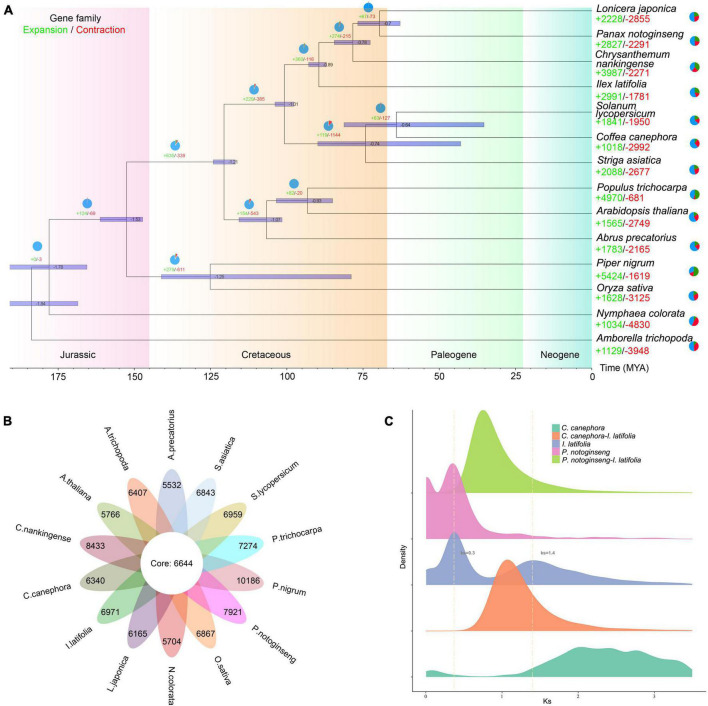
Genome evolution analysis of *Ilex latifolia*. **(A)** Expansion and contraction of gene families and phylogenetic relationships and divergence times between *I. latifolia* and other plant species. The light green numbers represent the numbers of expanded gene families, and the red numbers represent the numbers of contracted gene families. **(B)** The shared and unique gene families were compared between *I. latifolia* and 13 other angiosperm species. Each number represents the number of gene families. **(C)** Ks distribution and WGD events in *I. latifolia*. The Ks distribution of *I. latifolia* shows two peaks, one at approximately 0.3 (WGD 2) and another at approximately 1.4 (WGD 1).

Aquifoliales was stably recovered as the first diverging lineage of campanulids based on plastid data (Moore et al., 2010; Li et al., 2019); however, it repeatedly fell within lamiids in phylogenies reconstructed based on nuclear genes (Zeng et al., 2017; Yang et al., 2020). To resolve the phylogenetic position of Aquifoliales, we extracted 1,002 low-copy orthologous nuclear genes from 14 angiosperm species and reconstructed a highly supported phylogenetic tree (Figure 2A). Our results showed that the overall relationships of those 14 species were nearly identical to the backbone Angiosperm Phylogeny Group IV (APG IV, 2016), and *I. latifolia* was placed at the basal position of campanulids as the sister group to all other campanulids (BS = 100%), supporting the hypothesis that Aquifoliales belongs to campanulids. The divergence of Aquifoliales represent the initiation of campanulid differentiation, which was estimated to have started approximately 78 million years ago. Among all protein-coding genes, 6,644 gene families were shared by all 14 angiosperms, and 6,971 gene families were specific to *I. latifolia* (Figure 2B). In addition to the specific gene families, 2,991 gene families were found to have significantly expanded in *I. latifolia*, whereas 1,781 gene families significantly contracted (*p* < 0.05, Figure 2A). The specific and expanded gene families may have contributed to the development of the species-specific properties of this plant, leading to its distinct morphological, physiological, and genetic characteristics. GO analysis shows that the specific and expanded gene families in holly are associated with secondary metabolic processes, auxin catabolic process and other activities (Supplementary Figure 6), notably, KEGG analysis shows that both specific and expanded gene families related to terpene skeleton biosynthesis (Supplementary Figure 7).

Whole-genome duplication (WGD) events have commonly occurred among flowering plants (Van de Peer et al., 2017). To identify potential WGD events during the course of *I. latifolia* evolution, we calculated the Ks values of paralogs located in syntenic blocks of the *I. latifolia* genome. The resulting Ks distribution of *I. latifolia* showed two distinct peaks, one at approximately 0.3 (WGD 1) and the other at approximately 1.4 (WGD 2), suggesting that the ancestors of *I. latifolia* underwent at least two WGD events (Figure 2C and Supplementary Figures 8, 9). As studies of the other available genome from this genus, *I. polyneura*, have also identified two WGDs, with a similar Ks distribution pattern (Yao et al., 2022), we can speculate that the two WGDs likely occurred in the common ancestor of the two *Ilex* species. The more ancient WGD (WGD2) showed a peak before the divergence of *I. latifolia* and coffee, which would therefore correspond to the polyploidy event shared by all eudicots. The synteny analysis between *I. latifolia* and grape verified this conclusion by showing a 2:1 ratio of syntenic blocks (Supplementary Figure 9), which is expected to be a result of WGD1 in *I. latifolia*, and *I. latifolia* and grape should also share WGD2. The observation that the WGD2 peak of *I. latifolia* did not coincide with the peak in coffee can likely be explained by the differential evolutionary rates of the two species. For WGD1, although *Panax notoginseng* also showed a peak at 0.3, this Araliaceae species likely experienced an independent WGD, different from that in *I. latifolia*, because *P. notoginseng* and *I. latifolia* diverged earlier than the two independent WGDs. These findings suggest the occurrence of frequent and independent polyploidization events among different angiosperm lineages and the intensive occurrence of such events at certain geological times during angiosperm evolution.

### Biosynthesis of pentacyclic triterpenoid saponins

Triterpenes constitute a large and structurally diverse class of natural products with considerable industrial and pharmaceutical value. The biosynthetic process involves a series of enzymes. To date, the biosynthesis of precursors and the corresponding enzymes have been identified (Sawai and Saito, 2011; Miettinen et al., 2017). However, the final steps are much more complicated among different taxa. Three types of enzymes–Oxidosqualene cyclases (OSC), cytochrome P450 monooxygenases (CYP450) and uridine diphosphate-dependent glycosyltransferases (UGT) are considered to be involved in the final steps, among which, CYP450-catalyzed structural modifications are the most critical and determine the diversification and functionalization of the triterpene scaffolds (Ghosh, 2017). CYP450 is the largest family of enzymes involved in plant metabolism, and some of its members can catalyze the formation of derivatives from basic triterpene skeletons with modified structures and various functions. The members of the CYP51H, CYP71A, D, CYP72A, CYP81Q, CYP87D, CYP88D, L, CYP89A, CYP93E, CYP705A, CYP708A, and CYP716A, C, E, S, U, and Y subfamilies are reported to be responsible for the biosynthesis and structural modification of triterpenes and related derivatives (Miettinen et al., 2017; Zheng et al., 2019).

**FIGURE 3:**
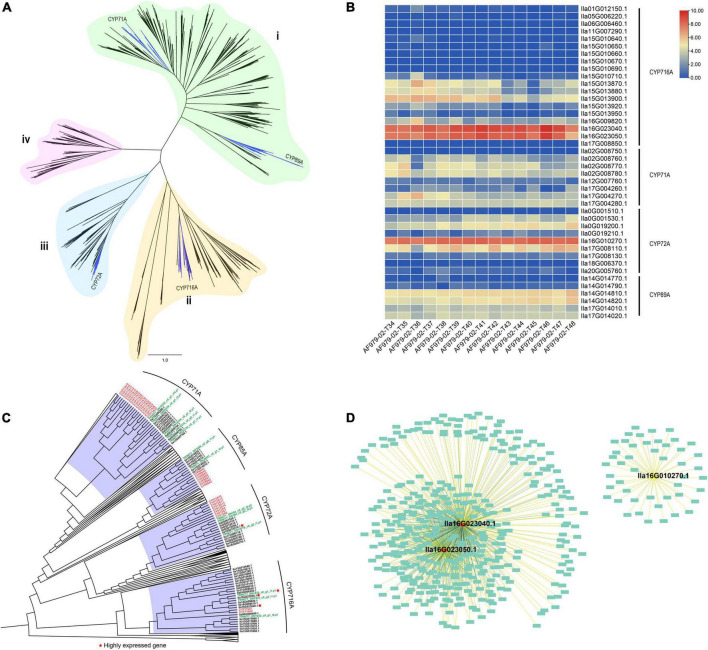
Transcriptomes of *Ilex latifolia* leaves at five developmental stages reveal the biosynthesis of pentacyclic triterpenoid saponins. **(A)** Phylogeny of 395 putative functional CYP450 genes from *I. latifolia*. **(B)** Heatmap of CYP450 gene expression related to triterpenoid synthesis. **(C)** A simplified CYP450 phylogenetic tree for *Amborella trichopoda*, *Arabidopsis thaliana*, *I. latifolia*, *I. cornuta*, and *Oryza sativa*. **(D)** Sub-network for three highly expressed CYP450 genes lla16G023040.1, lla16G023050.1, and lla16G010270.1.

To identify CYP450 genes involved in the biosynthesis of pentacyclic triterpenoid saponins in *I. latifolia*, we performed a genome-wide search for CYP450 genes in *I. latifolia*, *Amborella trichopoda*, *Arabidopsis thaliana*, *Oryza sativa*, and *P. notoginseng* by using BLAST and HMMER and conducted a phylogenetic analysis using the identified genes. The phylogenetic analysis indicated that all CYP450 genes could be classified into four monophyletic groups (i, ii, iii, and iv). The *I. latifolia* genome encodes 417 CYP450 genes, including four subfamilies with possible relationships to triterpene biosynthesis—CYP71A, CYP72A, CYP89A, and CYP716A—distributed in Groups i, ii, and iii (Figure 3A). Among the four subfamilies, three appear to have expanded compared with their numbers in *P. notoginseng* (another campanulid species): CYP71A (10 members), CYP72A (10 members), and CYP716A (22 members) (Supplementary Table 12). We further sequenced the transcriptomes of *I. latifolia* leaves at five developmental stages (three replicates for each stage) and examined the expression of these CYP450 genes using transcriptomic data. We found that two CYP716A genes (*Ila16G023040.1* and *Ila16G023050.1*) and one CYP72A gene (*Ila16G010270.1*) were always highly expressed at all five stages, suggesting that they probably play important roles in the biosynthetic pathway of the *I. latifolia* pentacyclic triterpenoid saponins (Figure 3B).

*Ilex latifolia* is preferentially used as the material for making Kudingcha over other species because of the higher levels of pentacyclic triterpenoid saponins levels in its leaves, and our metabolomics study also identified higher pentacyclic triterpenoid saponin concentrations in *I. latifolia* than in its close relative *I. cornuta* (Supplementary Figure 10). We infer that this difference should be attributed to both different CYP450 gene numbers and their differential expression in the two species (Supplementary Figure 11). There were significantly fewer CYP71A (5 members), CYP72A (3 members) and CYP716A (3 members) genes in *I. cornuta* (Supplementary Table 12) than in *I. latifolia*, and detailed observations revealed that no orthologs of the highly expressed *I. latifolia* CYP72A (*Ila16G010270.1*) and CYP716A (*Ila16G023040.1*) genes were detected in any of the *I. cornuta* transcriptomes (Figure 3C), suggesting the absence of these critical genes in *I. cornuta*. Therefore, it is likely that the absence of critical CPY450 genes in *I. cornuta* is responsible for the low pentacyclic triterpenoid saponin concentration in this species and explains why the leaves of *I. latifolia* are favored for the production of Kudingcha over other species in the same genus.

Coexpression analysis using the WGCNA approach was then conducted to identify more potential genes involved in the process. According to the observed expression patterns, *I. latifolia* genes were classified into 44 modules (Supplementary Figure 12). The highly expressed CYP716A and CYP72A genes were classified into two modules (black and light green). The two modules comprised 886 (black) and 369 (light green) genes showing similar expression patterns to CYP716A and CYP72A genes, respectively (Figure 3D). The GO analysis indicated that 41 genes in the black module were related to oxidoreductase activity, acting on paired donors with the incorporation or reduction of molecular oxygen (Supplementary Figure 13), and there were 47 genes in the light green module associated with metal ion binding (Supplementary Figure 14). Therefore, these genes are also likely to be involved in the biosynthesis of pentacyclic triterpenoid saponins.

### The biosynthesis of anthocyanidins regulates the red fruit color of *I. latifolia* and determines its ornamental value

**FIGURE 4:**
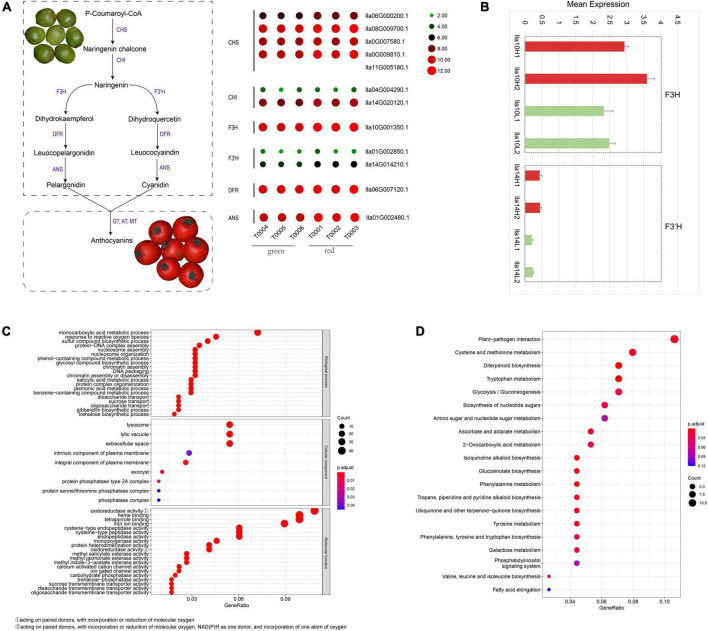
Anthocyanidin biosynthesis pathways in the *Ilex latifolia* fruit pericarp. **(A)** Genes in the biosynthesis pathway of pelargonidin and cyanidin and their expression in green and red fruit pericarps, respectively. **(B)** The expression of F3H (*Ila10G001350.1*) and F3’H (*Ila14G014210.1*) genes in the biosynthesis pathway of pelargonidin and cyanidin in the red (indicated by red histograms) and green (indicated by green histograms) pericarp using qRT–PCR analysis. **(C)** GO functional enrichment analysis of genes with significantly differential expression in *I. latifolia*. **(D)** KEGG analysis of genes with significantly differential expression in *I. latifolia*.

Red fruits are the most appreciated ornamental characteristic of *I. latifolia*. The red color of fruit pericarp is usually regulated by the synthesis of anthocyanidins, specifically the elevated synthesis of pelargonidin and cyanidin. To examine whether the green-to-red color change in *I. latifolia* fruits was related to the increased production of pelargonidin and cyanidin, we identified genes in the biosynthesis pathway of pelargonidin and cyanidin and examined their expression in green and red fruit pericarps, respectively. Most genes showed higher expression in red fruit pericarps than in green pericarps (Figure 4A and Supplementary Figure 15). To more precisely determine the expressional differences in these genes in the green and red fruit pericarps, we conducted an qRT–PCR analysis, and the results showed an average 1.07-fold increase in the expression of genes in the biosynthesis pathway of pelargonidin and cyanidin (Supplementary Figure 16), among which *F3H* (*Ila10G001350.1*) and *F3’H* (*Ila14G014210.1*) showed the largest increases in the red pericarp (>1.4-fold) (Figure 4B). These results indicate that the synthesis of pelargonidin and cyanidin plays an important role in the *I. latifolia* fruit pericarp color change.

In addition to the genes in the biosynthesis pathway of pelargonidin and cyanidin, other genes may also be related to the fruit pericarp color change in *I. latifolia*. We identified 161 genes with significantly differential expression in the transcriptomes of green and red fruit pericarps. GO analysis indicated that genes related to the “monocarboxylic acid metabolic process” term were the most enriched in the “biological process” category; “lysosome,” “lytic vacuole,” and “extracellular space” were most enriched terms in the “cellular component” category; and “oxidoreductase activity, acting on paired donors, with incorporation or reduction of molecular oxygen,” “heme binding,” “tetrapyrrole binding,” and “tetrapyrrole binding” were the most significant terms in the “molecular function” category (Figure 4C). KEGG analysis indicated that the maturation of the fruit pericarp is most tightly connected with the improvement of pathogen immunity, followed by metabolism, including cysteine and methionine metabolism, diterpenoid biosynthesis, tryptophan metabolism, and glycolysis/gluconeogenesis, suggesting a complex process of fruit pericarp development (Figure 4D).

## Discussion

The rapid development of genome sequencing technology has created the opportunity to acquire higher-quality genomes of important woody plant species with important ornamental and economic value. However, most of the angiosperm genomes sequenced to date have come from herbaceous plants (Kersey, 2019). Reference genomes for most woody plant families, which usually include important ornamental and economic tree species, are still lacking. Hollies, comprising more than 600 species worldwide, constitute one of the largest woody genera in Aquifoliaceae (Loizeau et al., 2016). However, research on these plants is currently hampered by the lack of reference genomes. In this study, we provide a chromosome-level assembly for *I. latifolia*, a species that can be used for beverage production, medicinal and ornamental purposes, and we used these data combined with multiomics data to explore the molecular basis underlying these features.

*Ilex latifolia* has multiple uses: its young leaves are employed for making healthy tea known as Kudingcha, and its evergreen leaves and red fruits are employed for ornamental purposes. The high-quality assembled genome of *I. latifolia* could provide much information for exploring questions related to the evolution of this species, the biosynthesis of the key components of Kudingcha, and the mechanism underlying its ornamental characteristics. Systematically, *I. latifolia* belongs to Aquifoliales, and this order mainly comprises woody trees and shrubs with sawtooth leaves and drupes. Our phylogenetic analysis recovered *I. latifolia* as the first diverging lineage of campanulids, consistent with the inferences made based on plastid genes (Moore et al., 2010; Li et al., 2019) but incongruent with the results based on nuclear genes (Zeng et al., 2017; Yang et al., 2020). Nevertheless, the branches splitting campanulids and lamiids are very short, suggesting that the campanulid-lamiid divergence was likely a rapid process and that Aquifoliales originated early, shortly after this divergence, as reflected in its own short branch length. Therefore, the plastid-nuclear conflict in the positioning of Aquifoliales should imply ancient hybridizations upon the early evolution of asterids, and the incongruence between our result and other studies based on nuclear genes may suggest potential incomplete lineage sorting. Hence, further evidence, such as genomic structures or phylogenies based on mitochondrial genes, is still required to conclusively determine the phylogenetic position of Aquifoliales.

The health care and medicinal effect of Kudingcha can likely be mainly attributed to the enrichment of pentacyclic triterpenoid saponins in the leaves of *I. latifolia*. Although the synthesis pathway of triterpenes has been well studied, the enzymes responsible for further modification leading to the generation of pentacyclic triterpenoid saponins remain poorly understood (Miettinen et al., 2017; Zheng et al., 2019). In this study, we screened potential enzymes based on a combination of genome-wide identification, phylogenomic analysis, and comparative transcriptomic approaches. Three members of the CYP450 superfamily belonging to the 72A and 716A subfamilies were suggested to be the most promising candidates according to phylogenomic clues and expressional patterns. Using the three candidate genes as hub genes, we performed WGCNA to identify more potential genes involved in the biosynthesis of pentacyclic triterpenoid saponins through coexpression networks. Our analytical strategy successfully identified two critical genes and a series of genes potentially involved in the biosynthesis of pentacyclic triterpenoid saponins. These identified genes will provide valuable information and will serve as targets for functional characterization in the future. Our study will greatly accelerate the elucidation of the whole biosynthesis pathway of this important metabolic product in *I. latifolia* and generate more interest in Kudingcha.

Anthocyanins found in plant organs commonly play an important role as an indicator of the ornamental value of plants (Li et al., 2020). As a fruit plant included in gardens, *I. latifolia* bears multiple red decorative berries in its ripening season and contributes to a very beautiful landscape. However, the mechanism of color change in *I. latifolia* fruit pericarps is still unknown. Therefore, the genes in the biosynthesis pathway of pelargonidin and cyanidin were identified in this study, and their expression in green and red fruit pericarps was examined. Interestingly, most of these genes were more highly expressed in red fruit pericarps than in green pericarps. This finding revealed that the synthesis of pelargonidin and cyanidin also plays an important role in the process of *I. latifolia* fruit pericarp color change. In addition, there were 161 genes with significantly differential expression in the transcriptomes of the two developmental stages of the fruit pericarp. Both GO and KEGG analyses indicate that fruit pericarp development is a complex process, and more studies are still needed to reveal the mechanism of fruit pericarp development.

In summary, the high-quality *I. latifolia* reference genome combined with transcriptome data provided insights into genome evolution, the biosynthesis of the pentacyclic saponin triterpenes found in Kudingcha, and the biosynthesis of anthocyanidins in the fruit pericarp of *I. latifolia*. The genome and transcriptome data obtained in this study will also be useful for studies concerning the production of holly teas and medicines, the mechanisms underlying the formation of important ornamental traits and molecular breeding in *I. latifolia* and other *Ilex* species.

## Data availability statement

The original contributions presented in the study are publicly available. This data can be found here: https://ngdc.cncb.ac.cn/gwh, GWHBIST00000000.

## Author contributions

K-WX, Y-FD, and J-YX conceived and designed the project. K-WX, C-XL, QZ, MZ, PZ, Y-MF, and Y-FD collected and generated the plant materials. K-WX, MZ, and X-FW performed all the data analyses under the supervision of J-YX, Y-FD, and Y-MF. K-WX, X-FW, and J-YX drafted the manuscript. All authors contributed to and approved the final manuscript.

## Footnotes

1
https://github.com/ruanjue/smartdenovo

2
https://github.com/nservant/HiC-Pro

3
http://www.timetree.org

4
https://github.com/TransDecoder/TransDecoder/releases

5
http://www.Arabidopsis.org/

6
https://www.ncbi.nlm.nih.gov/

7
http://tree.bio.ed.ac.uk/software/figtree
