# Supplementary material for: The chromosome-level holly (Ilex latifolia) genome reveals key enzymes in triterpenoid saponin biosynthesis and fruit color change
Source: Front Plant Sci. 2022 Aug 22;13:982323. doi: 10.3389/fpls.2022.982323 (PMC9441949; doi:10.3389/fpls.2022.982323)
Supplement: Supplementary file 1 [file Data_Sheet_1.docx]

**The chromosome-level holly (*Ilex latifolia*) genome reveals key enzymes in triterpenoid saponin biosynthesis and fruit colour change**

Ke-Wang Xu1,†, Xue-Fen Wei2,†, Chen-Xue Lin1, Min Zhang1, Min Zhang3, Qiang Zhang1, Peng Zhou3, Yan-Min Fang1, Jia-Yu Xue2,* & Yi-Fan Duan1,*

**Supplementary Tables:**

**Supplementary Table 1.** The statistics of raw sequencing data from Illumina sequencing.

| **Library** | **Data (Gb)** | **Depth (×)** | **Q20 (%)** | **Q30 (%)** |
| --- | --- | --- | --- | --- |
| 350 bp_1 | 44.31 | 57.35 | 97.60 | 93.34 |
| 350 bp_2 | 44.31 | 57.35 | 97.60 | 93.34 |
| Total | 88.62 | 114.71 | --- | --- |

**Supplementary Table 2.** The completeness assessment of core eukaryotic genes (CEG) in *Ilex latifolia* genome assemblies using CEGMA v2.5 based on CEGMA database.

| **Species** | **Number of 458 CEG* present in the assembly** | **Percentage of 458 CEGs present in the assembly** | **Number of 248 highly conserved CEGs present in the assembly** | **Percentage of 248 highly conserved CEGs present in the assembly** |
| --- | --- | --- | --- | --- |
| *I. latifolia* | 433 | 94.54% | 211 | 85.08% |

**Supplementary Table 3.** Completeness and statistics of assembly in the *Ilex latifolia* genomes.

| **Complete BUSCOs** | **Complete and single-copy BUSCOs** | **Complete and duplicated BUSCOs** | **Fragmented BUSCOs** | **Missing BUSCOs** | **Total Lineage BUSCOs** |
| --- | --- | --- | --- | --- | --- |
| 1,328 (92.22%) | 1,157 (80.35%) | 171 (11.88%) | 25 (1.74%) | 87 (6.04%) | 1,440 |

**Supplementary Table 4.** The statistical results of Hi-C assembly.

|  | **Scaffold** | **Contig** |
| --- | --- | --- |
| Number >1000 bp | 355 | 1,100 |
| Length >1000 bp (bp) | 766,017,350 | 765,942,850 |
| N50 >1000 bp (bp) | 33,446,872 | 1,263,541 |
| N90 >1000 bp (bp) | 24,069,099 | 309,942 |
| Max length | 56,602,566 | 6,818,015 |

**Supplementary Table 5.** Chromosome length of *Ilex latifolia* by Hi-C assembly.

| **Group** | **Cluster Num** | **Cluster Len** | **Order Num** | **Order Len** |
| --- | --- | --- | --- | --- |
| LG01 | 56 | 30,612,630 | 37 | 27,875,519 |
| LG02 | 27 | 23,794,532 | 18 | 22,707,065 |
| LG03 | 58 | 30,255,154 | 40 | 27,486,786 |
| LG04 | 53 | 27,325,022 | 31 | 24,066,099 |
| LG05 | 29 | 33,612,184 | 27 | 33,444,272 |
| LG06 | 42 | 39,541,207 | 33 | 38,093,649 |
| LG07 | 61 | 30,613,564 | 40 | 26,665,288 |
| LG08 | 82 | 41,098,777 | 52 | 35,912,234 |
| LG09 | 46 | 39,251,682 | 33 | 37,265,274 |
| LG10 | 58 | 31,737,923 | 36 | 28,817,853 |
| LG11 | 69 | 33,627,125 | 41 | 28,680,378 |
| LG12 | 29 | 33,824,559 | 23 | 32,624,361 |
| LG13 | 32 | 29,975,603 | 27 | 29,201,461 |
| LG14 | 90 | 63,105,569 | 56 | 56,597,066 |
| LG15 | 80 | 58,928,186 | 59 | 56,210,523 |
| LG16 | 82 | 56,855,462 | 58 | 53,276,681 |
| LG17 | 69 | 51,018,767 | 54 | 48,770,880 |
| LG18 | 55 | 43,054,838 | 42 | 41,125,091 |
| LG19 | 58 | 33,470,655 | 41 | 31,542,513 |
| LG20 | 19 | 32,741,317 | 17 | 32,482,553 |
| Total (Ratio %) | 1095 (99.55) | 764444756 (99.8) | 765 (69.86) | 712845546 (93.25) |

**Supplementary Table 6.** Statistics of repeat sequences in *I. latifolia*.

| **Type** | **Number** | **Length** | **Rate (%)** |
| --- | --- | --- | --- |
| ClassI/DIRS | 15,391 | 33,657,558 | 4.39 |
| ClassI/LINE | 3,157 | 6,491,791 | 0.85 |
| ClassI/LTR | 928 | 1,678,915 | 0.22 |
| ClassI/LTR/Copia | 30,434 | 46,768,235 | 6.11 |
| ClassI/LTR/Gypsy | 99,927 | 225,251,981 | 29.41 |
| ClassI/LTR\|DIRS | 21 | 22,064 | 0.00 |
| ClassI/PLE\|LARD | 32,017 | 53,091,294 | 6.93 |
| ClassI/SINE | 6,562 | 1,243,609 | 0.16 |
| ClassI/TRIM | 5,435 | 2,284,502 | 0.30 |
| ClassI/Unknown | 451 | 122,596 | 0.02 |
| ClassII/Crypton | 5 | 228 | 0.00 |
| ClassII/Helitron | 17,711 | 6,391,710 | 0.83 |
| ClassII/MITE | 2,672 | 587,279 | 0.08 |
| ClassII/Maverick | 1,802 | 811,676 | 0.11 |
| ClassII/TIR | 38,084 | 21,773,337 | 2.84 |
| ClassII/Unknown | 5,341 | 1,687,402 | 0.22 |
| PotentialHostGene | 41,796 | 10,028,617 | 1.31 |
| Total with overlap | 509,773 | 467,750,524 | 61.07 |
| Total without overlap | 509,773 | 406,033,919 | 53.01 |

**Supplementary Table 7.** The prediction of gene structures of *I. latifolia*.

| **Method** | **Software** | **Species** | **Gene number** |
| --- | --- | --- | --- |
| Ab initio | Genscan | - | 40,370 |
|  | Augustus | - | 42,103 |
|  | GlimmerHMM | - | 56,123 |
|  | GeneID | - | 68,683 |
|  | SNAP | - | 64,644 |
| Homology-based | GeMoMa | *Arabidopsis_thaliana* | 28,393 |
|  |  | *Artemisia_annua* | 37,468 |
|  |  | *Mikania_micranth* | 36,451 |
|  |  | *Helianthus_annuus* | 40,430 |
| RNAseq | TransDecoder | - | 84,433 |
|  | GeneMarkS-T | - | 51,871 |
|  | PASA | - | 44,184 |
| Integration | EVM | - | 35,218 |

**Supplementary Table 8.** The prediction results of gene structures of *I. latifolia*.

| **Gene set** | **Index value** |
| --- | --- |
| Gene Number | 35,218 |
| Total gene length | 196,713,679 |
| Average Gene length | 5,585.60 |
| Total exon length | 54,836,840 |
| Average exon length | 1,557.07 |
| Exon number | 181,301 |
| Average exon number | 5.15 |
| Total CDS length | 42,499,685 |
| Average CDS length | 1,206.76 |
| CDS Number | 174,550 |
| Average CDS Number | 4.96 |
| Intron length | 141,876,839 |
| Average intron length | 4,028.53 |
| Intron number | 146,083 |
| Average intron number | 4.15 |

**Supplementary Table 9.** The statistical results of functional annotation.

| **Annotation database** | **Annotated number** | **Percentage (%)** |
| --- | --- | --- |
| GO_Annotation | 18,922 | 53.73 |
| KEGG_Annotation | 11,169 | 31.71 |
| KOG_Annotation | 18,721 | 53.16 |
| TrEMBL_Annotation | 32,738 | 92.96 |
| nr_Annotation | 32,778 | 93.07 |
| All_Annotated | 32,814 | 93.17 |

## Supplementary Table 10. *K*-mer statistics of genome sequencing results from *I. latifolia.*

| **Statistical indices** | **Index value** |
| --- | --- |
| *K*-mer | 21 |
| *K*-mer number | 71,218,217,963 |
| *K*-mer Depth | 89 |
| Genome size (Mbp) | 772.55 |
| Repeat sequence (%) | 47.59 |
| Heterozygosity (%) | 0.85% |
| GC content (%) | 37.60 |

**Supplementary Table 11.** Statistical summaries of the genome assembly of *Ilex latifolia*.

| **Contig number** | **Contig length(bp)** | **Contig N50(bp)** | **Contig N90(bp)** | **Contig max (bp)** | **GC content(%)** | **Gap total length(bp)** |
| --- | --- | --- | --- | --- | --- | --- |
| 844 | 765,942,850 | 1,464,319 | 462,314 | 6,818,015 | 36.44 | 0 |

| **Category** | ***I. latifolia*** | ***I. cornuta*** | ***P. notoginseng*** | ***A. thaliana*** |
| --- | --- | --- | --- | --- |
| CYP71A | 10 | 5 | 3 | 16 |
| CYP716A | 22 | 3 | 7 | 2 |
| CYP72A | 10 | 3 | 10 | 8 |
| CYP89A | 6 | 2 | 4 | 7 |

**Supplementary Table 12.** The gene number of four subfamilies of CYP450 in *Ilex latifolia*, *I. cornuta*, *P. notoginseng*, and *A. thaliana*.
