# Supplementary material for: The chromosome-level holly (Ilex latifolia) genome reveals key enzymes in triterpenoid saponin biosynthesis and fruit color change
Source: Front Plant Sci. 2022 Aug 22;13:982323. doi: 10.3389/fpls.2022.982323 (PMC9441949; doi:10.3389/fpls.2022.982323)
Supplement: Supplementary file 2 [file Data_Sheet_2.docx]

**The chromosome-level holly (*Ilex latifolia*) genome reveals key enzymes in triterpenoid saponin biosynthesis and fruit colour change**

Ke-Wang Xu1,†, Xue-Fen Wei2,†, Chen-Xue Lin1, Min Zhang1, Min Zhang3, Qiang Zhang1, Peng Zhou3, Yan-Min Fang1, Jia-Yu Xue2,* & Yi-Fan Duan1,*

**Supplementary Figures:**


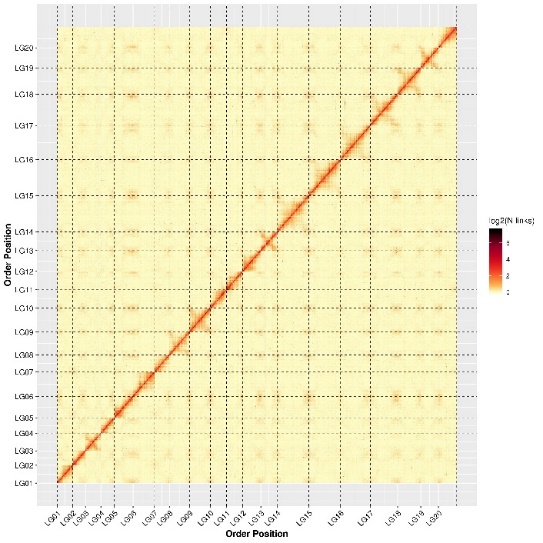


## Supplementary Figure 1. Genome-wide chromatin interaction signal heatmap of *Ilex latifolia* genome.


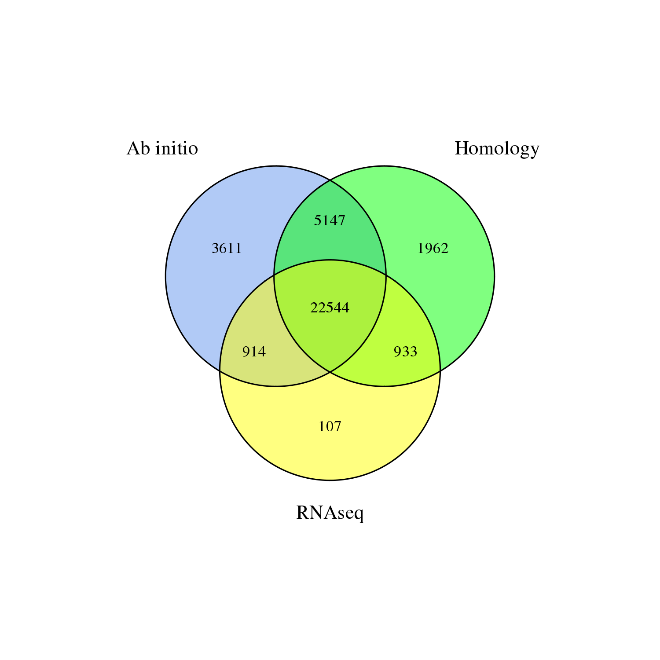


**Supplementary Figure 2.** de novo gene predictions of *Ilex latifolia* genome based on three different methods.


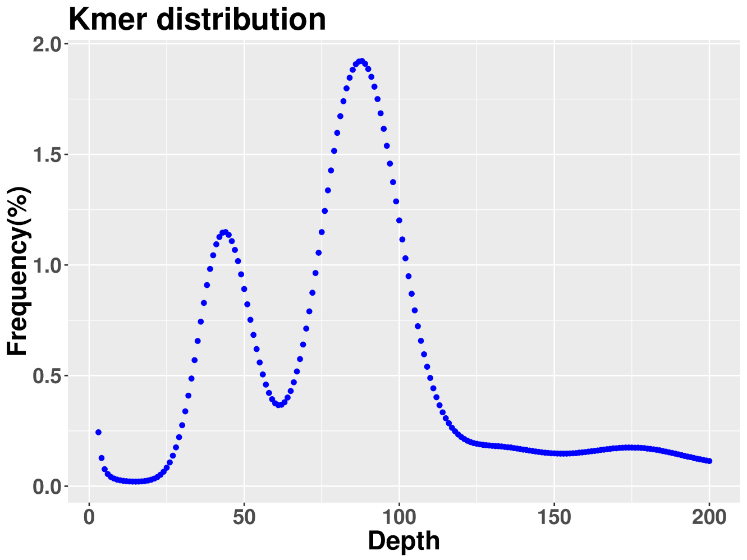
**Supplementary Figure 3.** Estimation of *Ilex latifolia* genome size using Jellyfish with a 21 K-mer distribution.

**
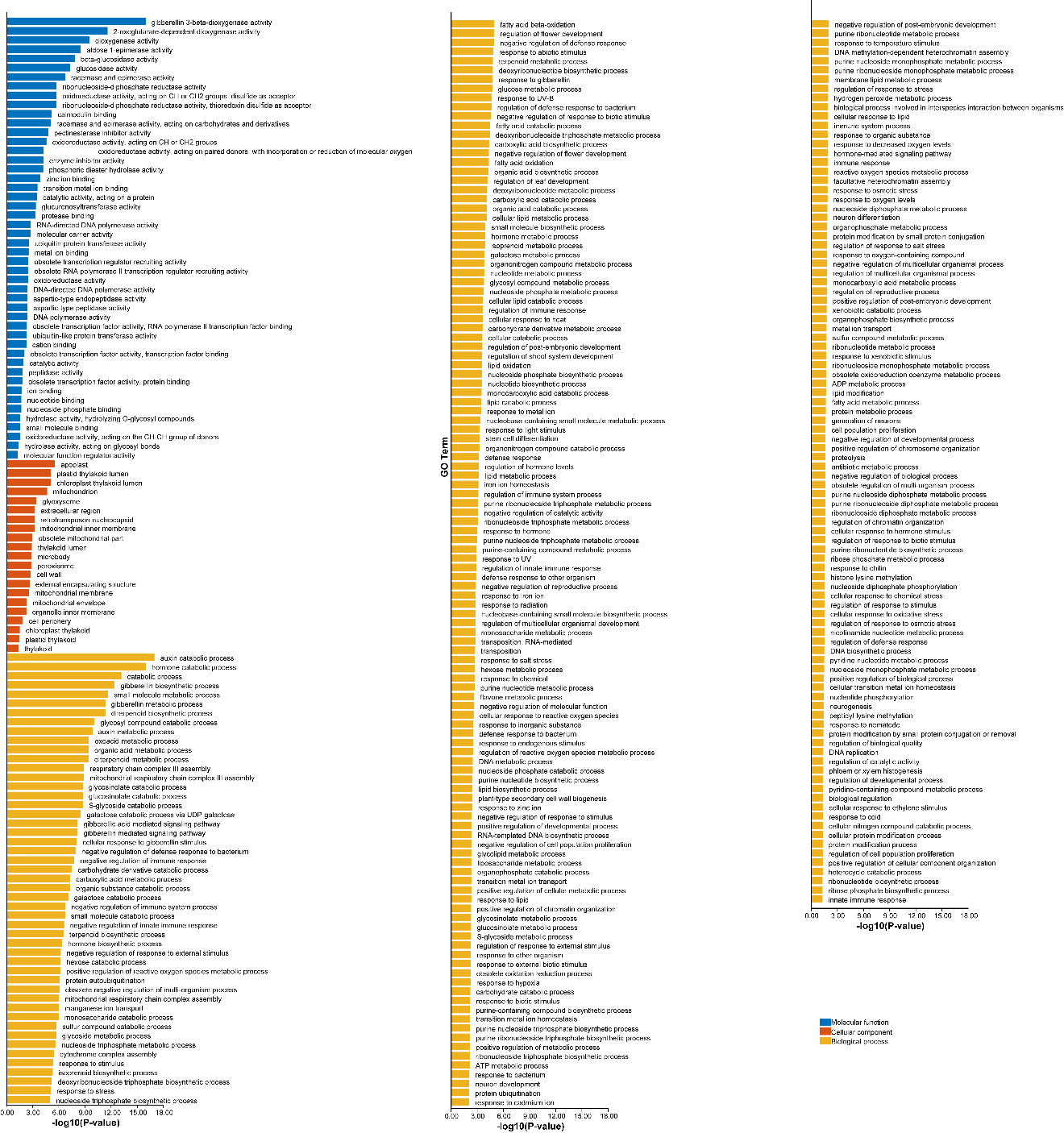
**

**Supplementary Figure 4.** Specific gene families were analyzed for GO enrichment.

**
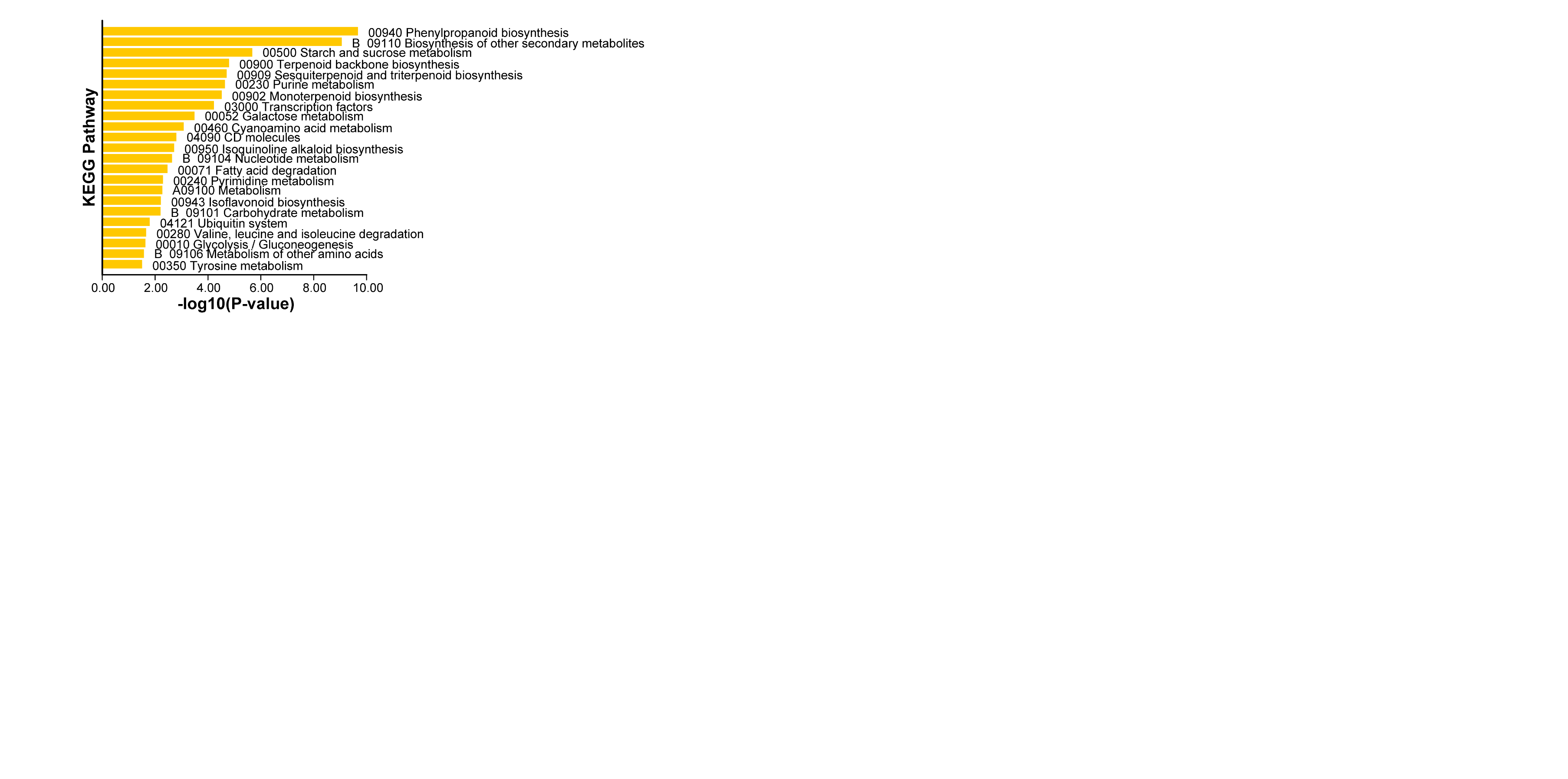
**

**Supplementary Figure 5.** Specific gene families were analyzed for KEGG enrichment.


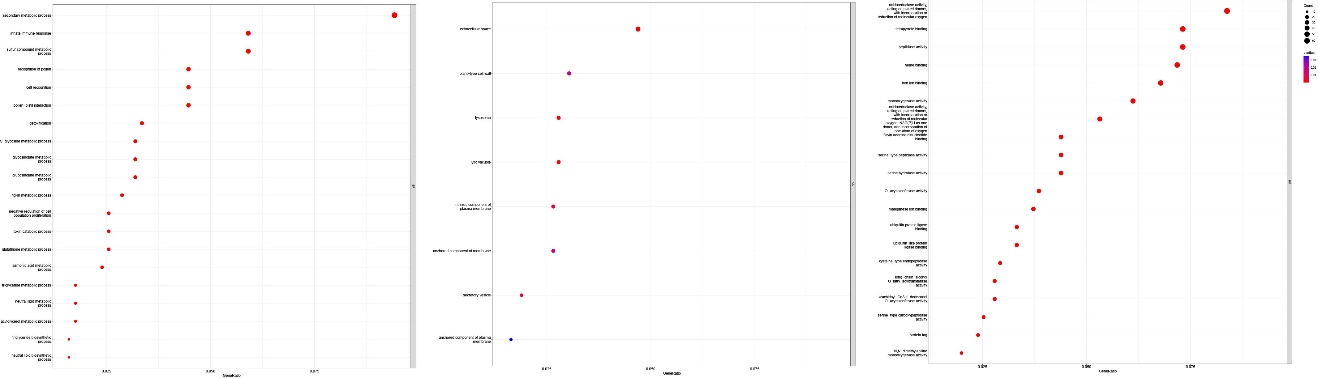


**Supplementary Figure 6.** GO analysis shows that the specific and expanded gene families in holly are associated with secondary metabolic processes, auxin catabolic process and other activities.


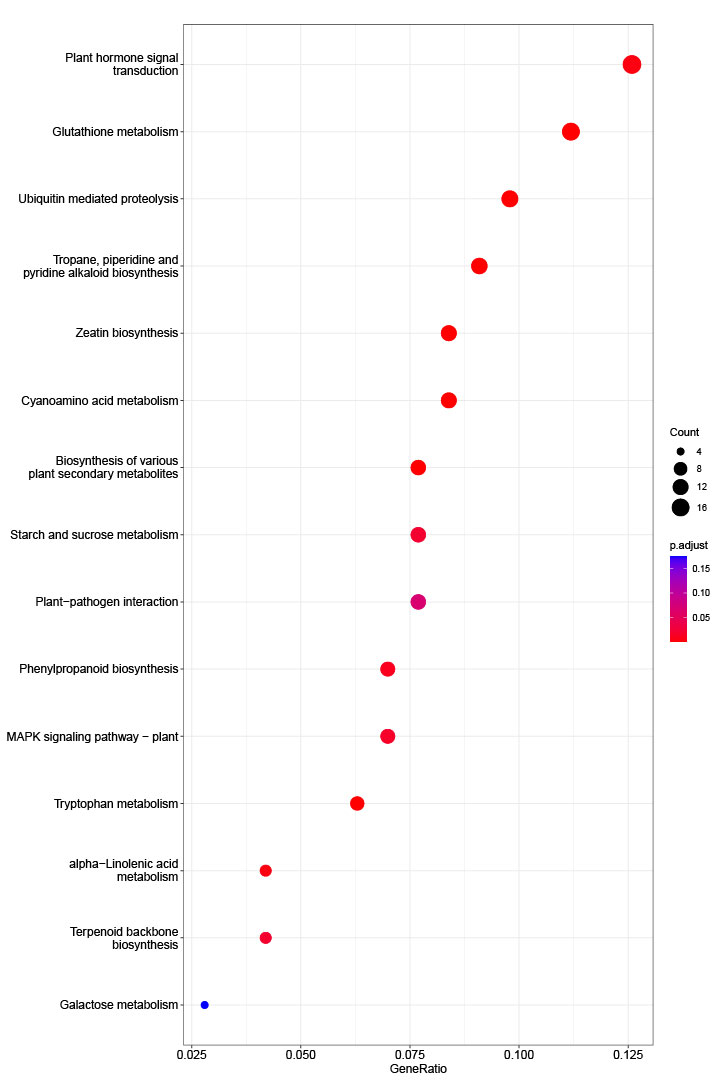


**Supplementary Figure 7.** KEGG analysis shows that both specific and expanded gene families related to terpene skeleton biosynthesis.

**
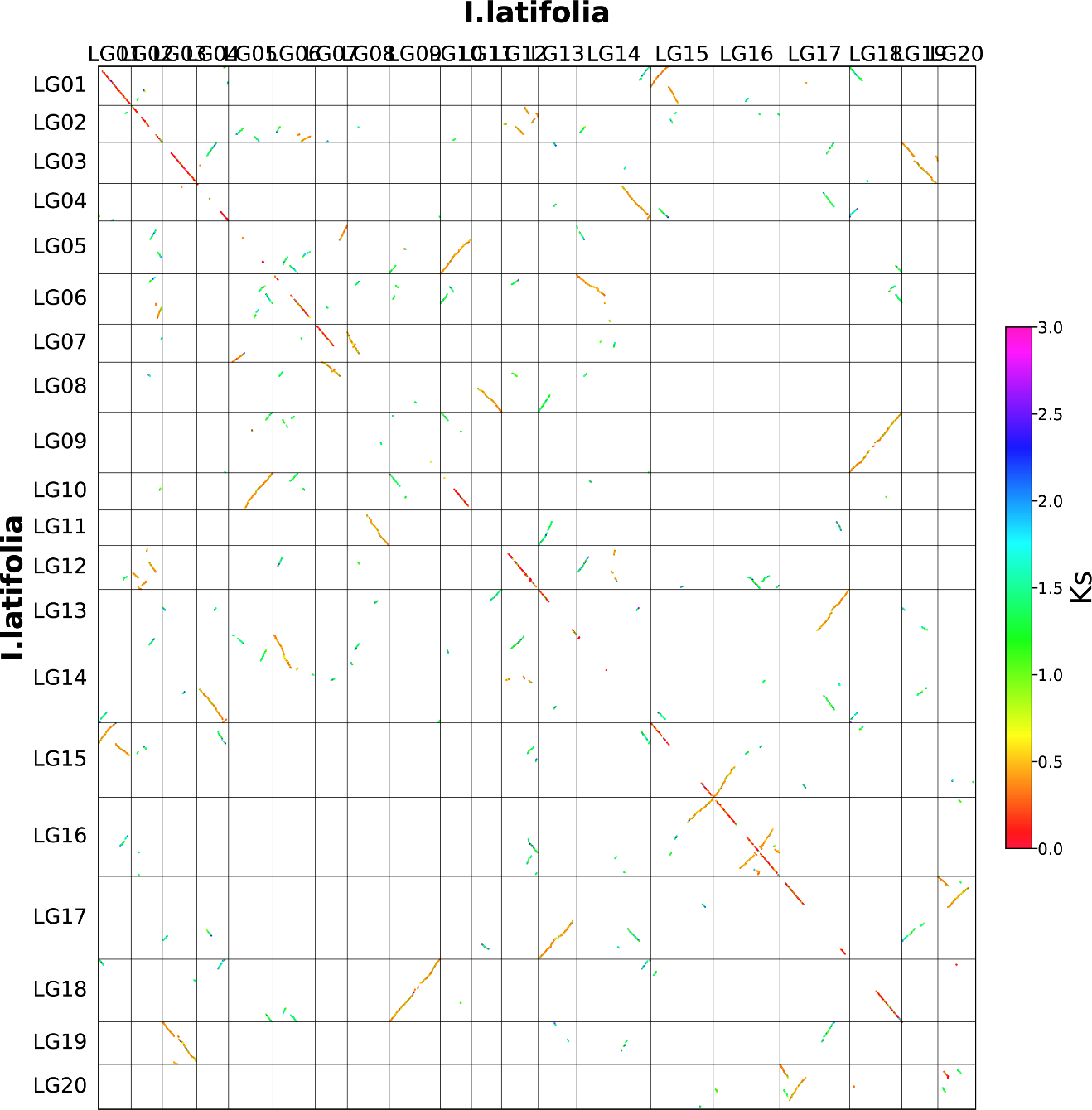
Supplementary Figure 8.** Syntenic dot plot of the intragenomic comparison of *I. latifolia*.


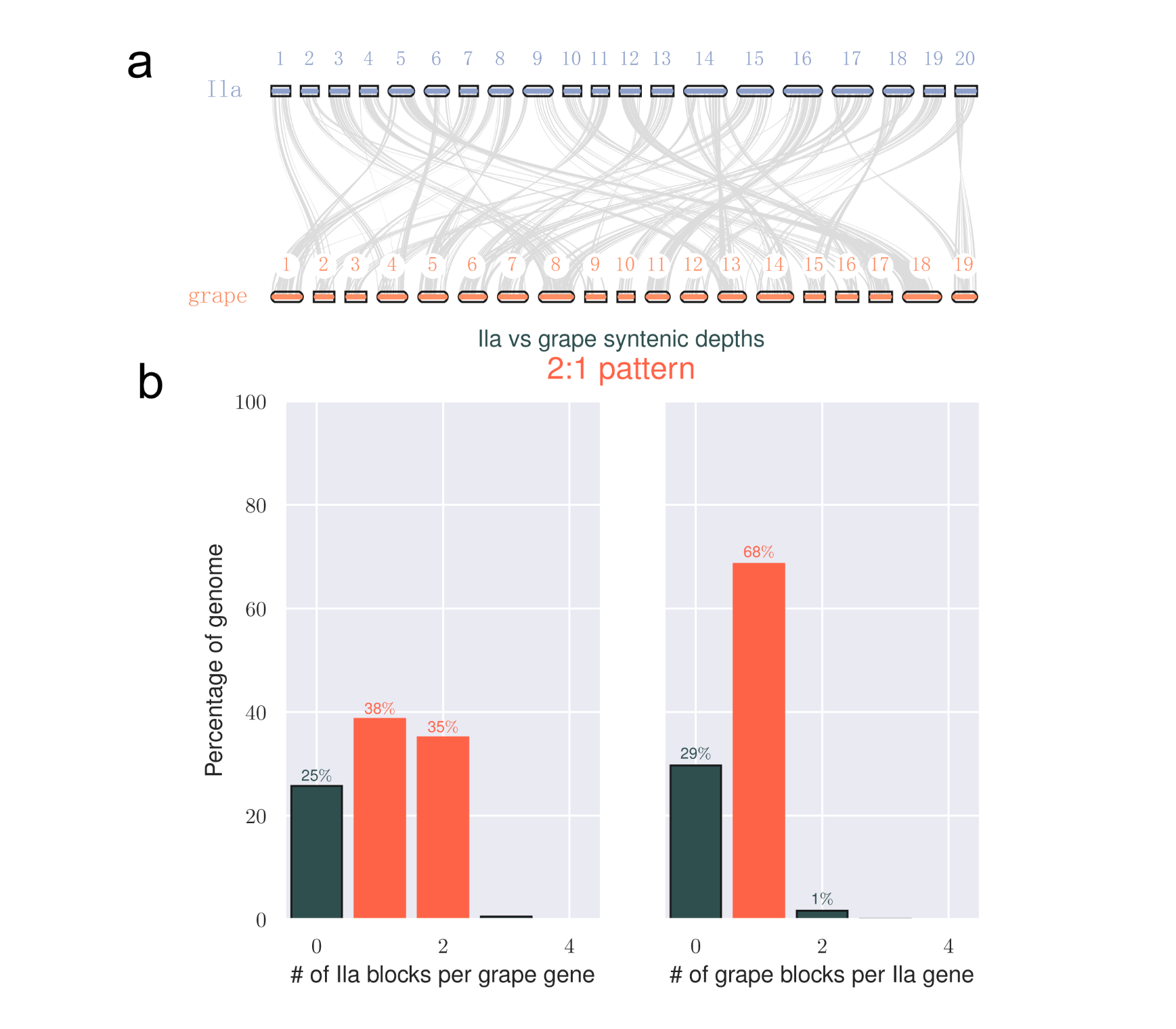


**Supplementary Figure 9. a** Syntenic comparison between *Ilex latifolia* and *Vitis vinifera*. Syntenic blocks were linked by gray lines. **b** Percentage of *Ilex latifolia* blocks per grape gene and percentage of grape blocks per *Ilex latifolia* gene.


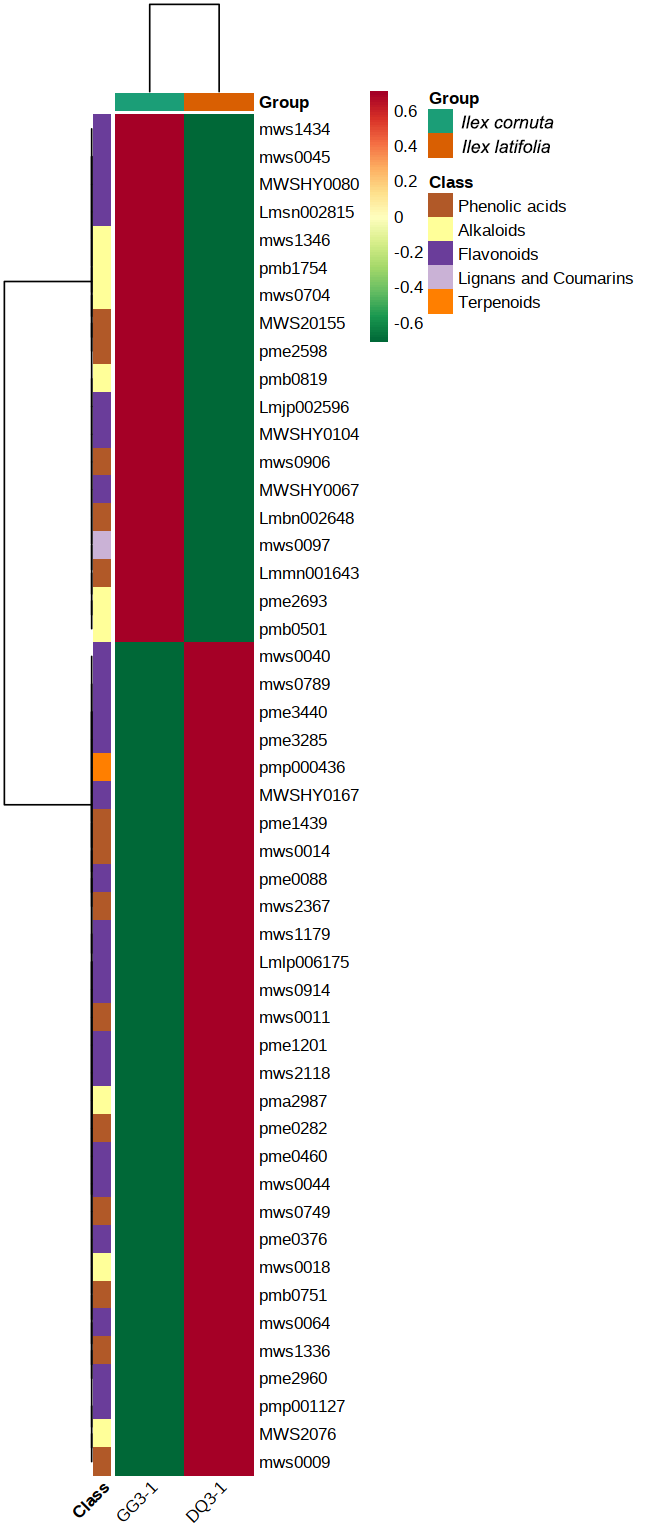


**Supplementary Figure 10.** Cluster diagram of differential metabolites of KEGG pathways.

**
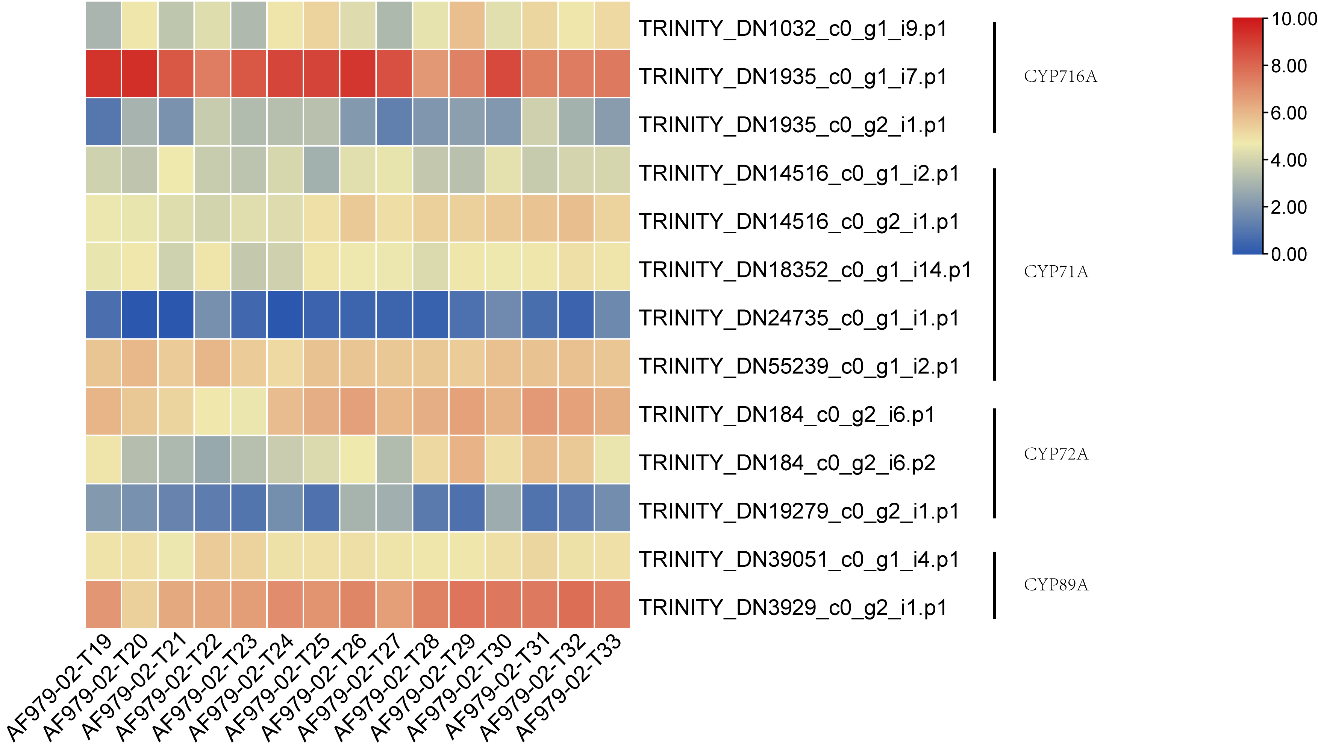
**

**Supplementary Figure 11.** Heatmap of CYP450 gene expression related to triterpenoid synthesis in *Ilex cornuta*


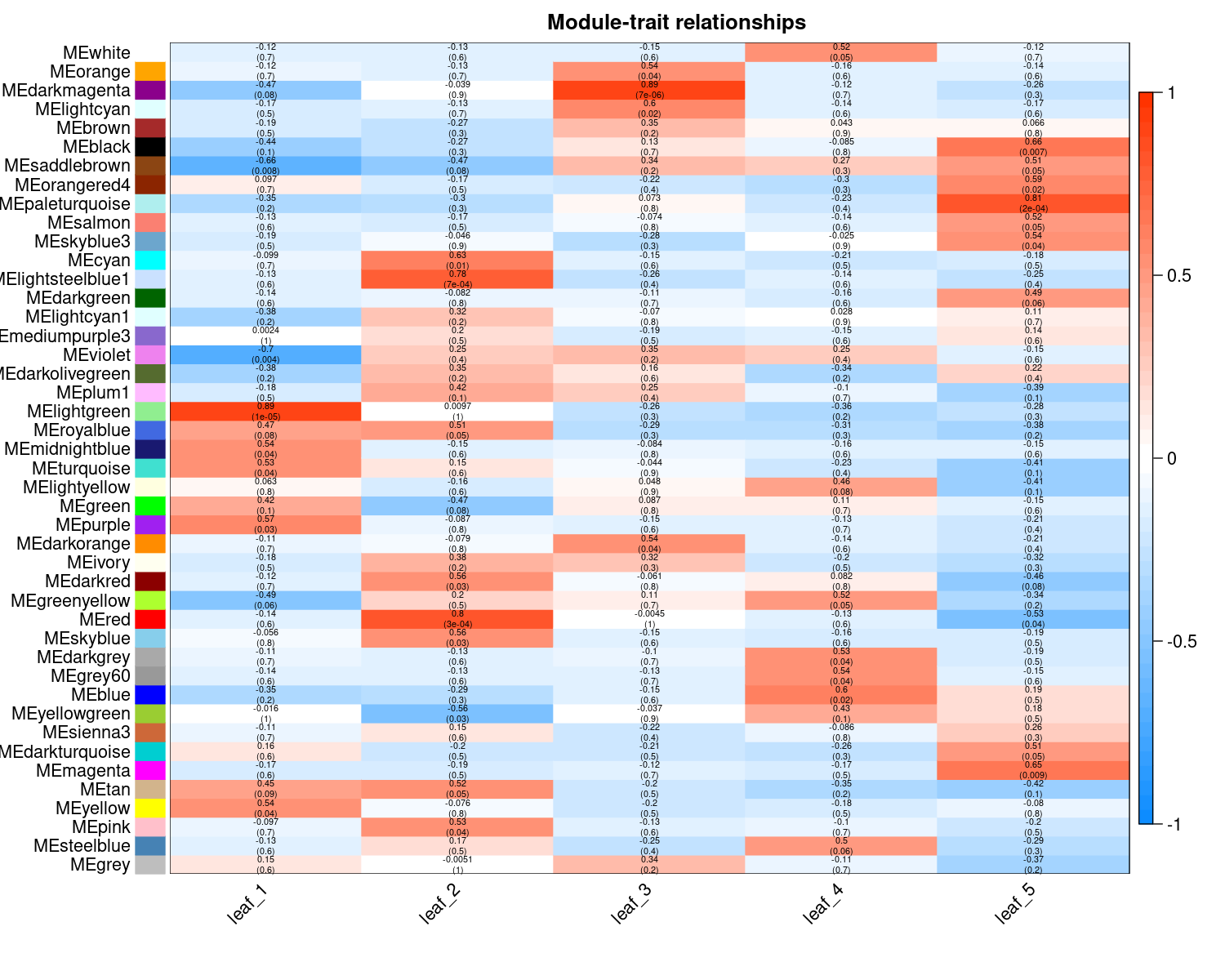
**Supplementary Figure 12.** WGCNA analysis in *Ilex latifolia* leaf tissues showing the module-tissue association. Rows correspond to 44 modules. A high degree of correlation between a specific module and the tissue type was indicated by dark red.

**
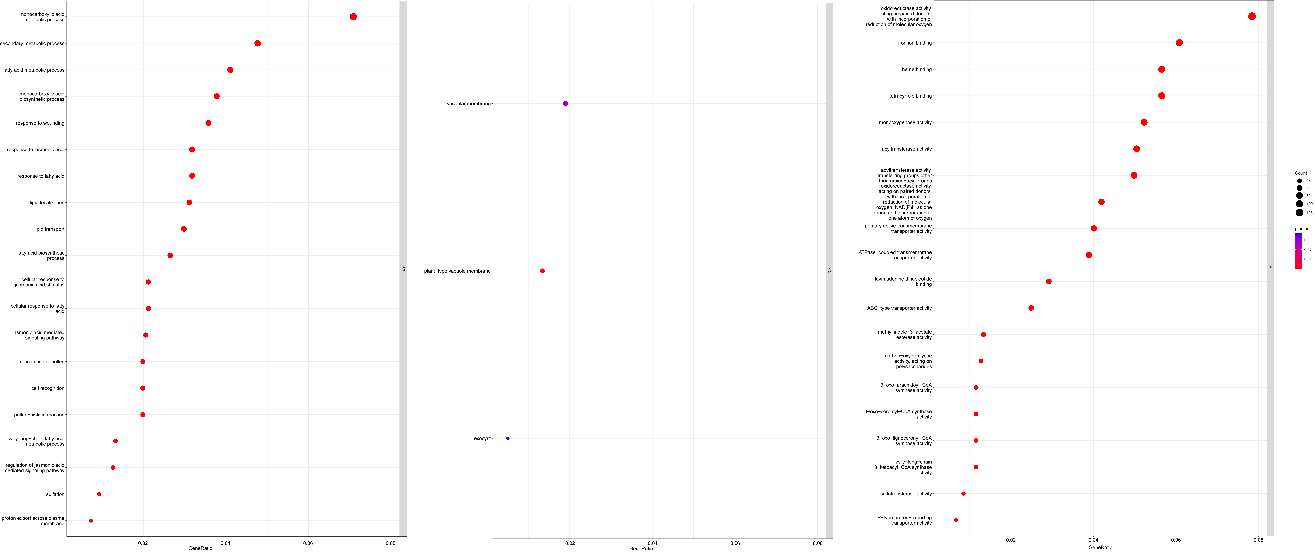
**

**Supplementary Figure 13.** The GO analysis of genes in the black module.

**
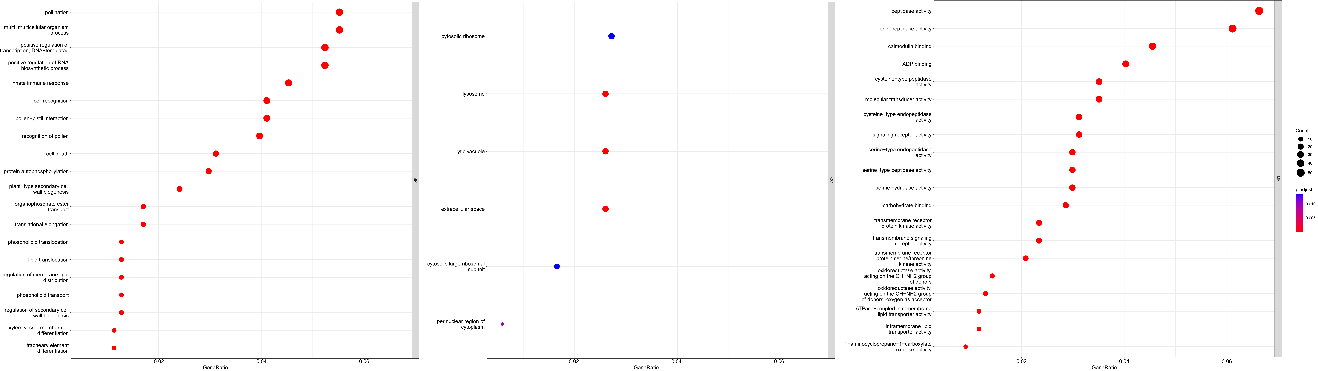
**

**Supplementary Figure 14.** The GO analysis of genes in the light green module.

**
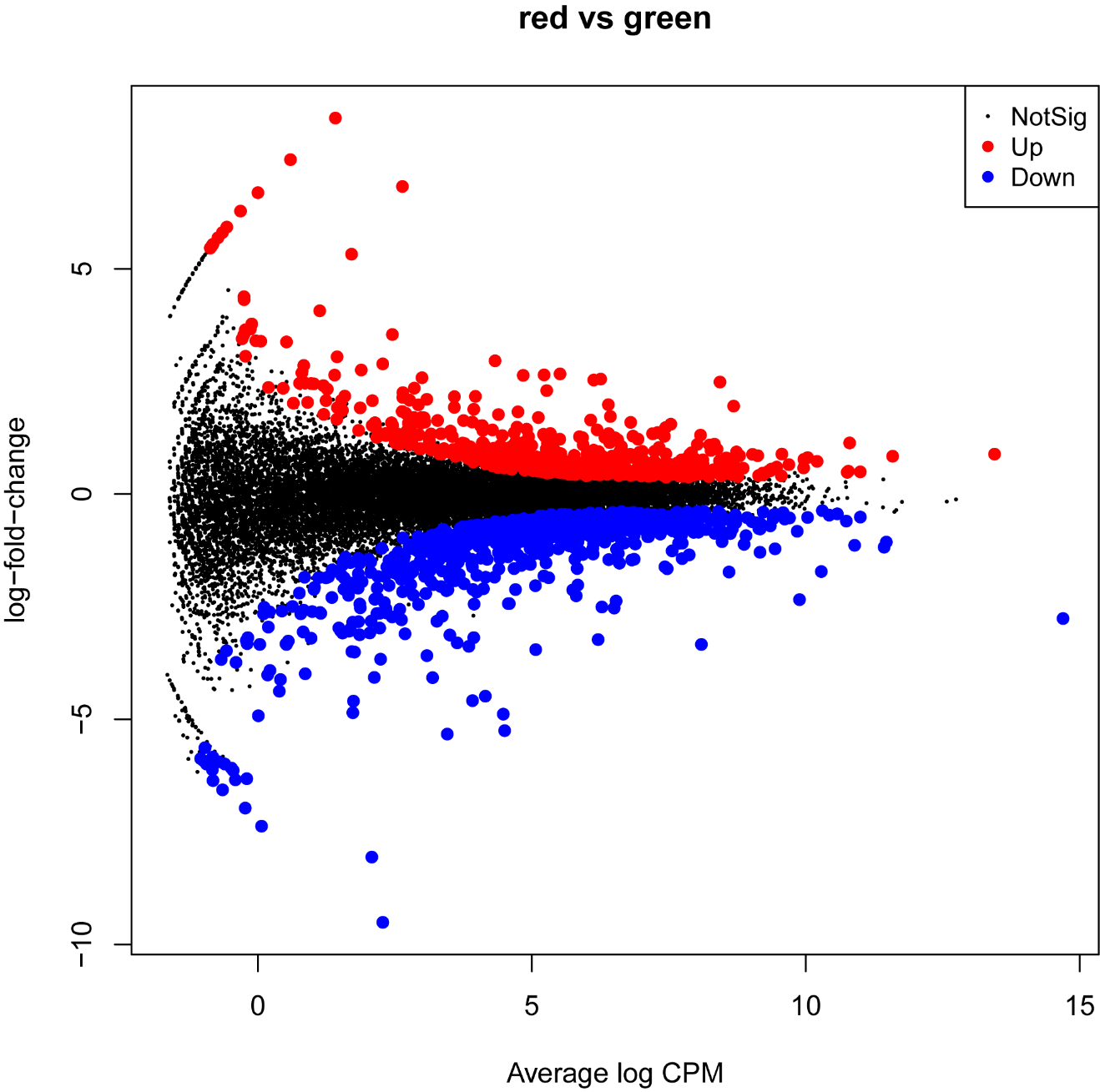
**

**Supplementary Figure 15.** Volcano map of differentially expressed genes in green and red fruit pericarps of *Ilex latifolia*.


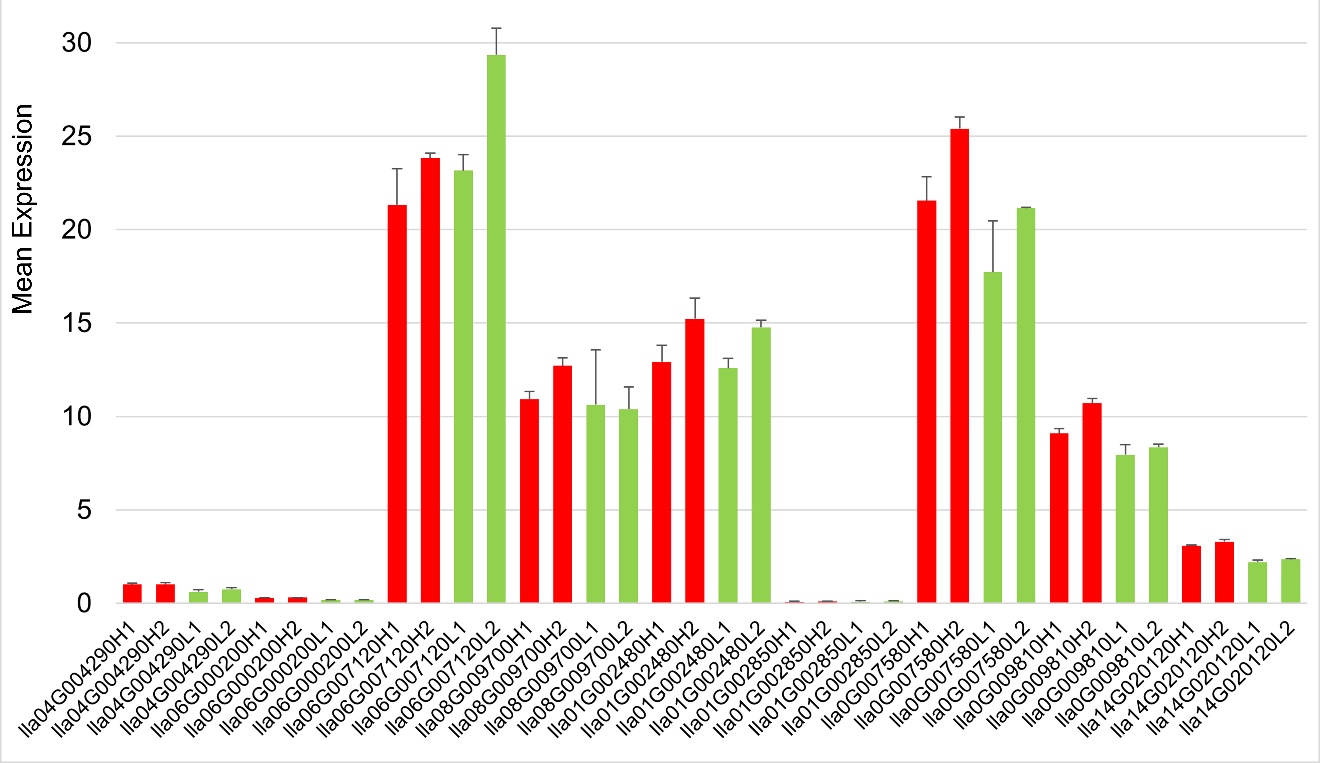


**Supplementary Figure 16.** The expression of those genes in the biosynthesis pathway of pelargonidin and cyanidin in the red (indicated by red histograms) and green (indicated by green histograms) pericarp using qRT–PCR analysis.
